# Supplementary material for: Hybrid Models and Biological Model Reduction with PyDSTool
Source: PLoS Comput Biol. 2012 Aug 9;8(8):e1002628. doi: 10.1371/journal.pcbi.1002628 (PMC3415397; doi:10.1371/journal.pcbi.1002628)
Supplement: Text S4 — Complete source code for the PyDSTool package (version 0.88.120504). Includes API documentation and help files linking to web pages. This file is identical to the current public release on Sourceforge.net. (ZIP) [file pcbi.1002628.s004.zip › PyDSTool/html/PyDSTool.Toolbox.optimizers.line_search.golden_section-pysrc.html]

xml version="1.0" encoding="ascii"?


PyDSTool.Toolbox.optimizers.line\_search.golden\_section


| Home | Trees | Indices | Help | PyDSTool |
| --- | --- | --- | --- | --- |

Package PyDSTool ::
Package Toolbox ::
Package optimizers ::
Package line\_search ::
Module golden\_section

[hide private]

[frames] | no frames]

# Source Code for Module PyDSTool.Toolbox.optimizers.line\_search.golden\_section

```
 1   
 2  # Matthieu Brucher 
 3  # Last Change : 2007-08-26 19:50 
 4   
 5  """ 
 6  Line Search with the golden section method 
 7  """ 
 8   
 9  import math 
10   


11 -class GoldenSectionSearch(object):


12    """ 
13    Line Search with the golden section method 
14    """ 


15 -  def __init__(self, min_alpha_step, alpha_step = 1., **kwargs):


16      """ 
17      Needs to have : 
18        - a minimum step size (min_alpha_step) 
19      Can have : 
20        - a step modifier, a factor to modulate the step (alpha_step = 1.) 
21      """ 
22      self.minStepSize = min_alpha_step 
23      self.stepSize = alpha_step 
24      self.goldenNumber = (math.sqrt(5) - 1) / 2.

25   


26 -  def __call__(self, origin, function, state, **kwargs):


27      """ 
28      Returns a good candidate 
29      Parameters : 
30        - origin is the origin of the search 
31        - function is the function to minimize 
32        - state is the state of the optimizer 
33      """ 
34      direction = state['direction'] 
35      ak = 0 
36      v_ak = function(origin + ak * direction) 
37      if 'initial_alpha_step' in state: 
38        bk = state['initial_alpha_step'] 
39      else: 
40        bk = self.stepSize 
41      v_bk = function(origin + bk * direction) 
42   
43      uk = ak + self.goldenNumber * (bk - ak) 
44      v_uk = function(origin + uk * direction) 
45      lk = ak + (1 - self.goldenNumber) * (bk - ak) 
46      v_lk = function(origin + lk * direction) 
47   
48      while True: 
49        if v_uk < v_lk: 
50          if (bk - lk) < self.minStepSize: 
51            state['alpha_step'] = uk 
52            return origin + uk * direction 
53          ak = lk 
54          v_ak = v_lk 
55          lk = uk 
56          v_lk = v_uk 
57          uk = ak + self.goldenNumber * (bk - ak) 
58          v_uk = function(origin + uk * direction) 
59        else: 
60          if (uk - ak) < self.minStepSize: 
61            state['alpha_step'] = lk 
62            return origin + lk * direction 
63          bk = uk 
64          v_bk = v_uk 
65          uk = lk 
66          v_uk = v_lk 
67          lk = ak + (1 - self.goldenNumber) * (bk - ak) 
68          v_lk = function(origin + lk * direction)

69
```

  


| Home | Trees | Indices | Help | PyDSTool |
| --- | --- | --- | --- | --- |

|  |  |
| --- | --- |
| Generated by Epydoc 3.0.1 on Fri May 4 15:24:21 2012 | http://epydoc.sourceforge.net |
